# Acute Toxicity With Lactic Acidosis Associated With Cannabinoid Gummies

**DOI:** 10.7759/cureus.72495

**Published:** 2024-10-27

**Authors:** Meagan Sheldon, Michael Ammons, Kenneth Nugent

**Affiliations:** 1 Internal Medicine/Pulmonary and Critical Care Medicine, Texas Tech University Health Sciences Center, Lubbock, USA; 2 Internal Medicine, Texas Tech University Health Sciences Center, Lubbock, USA

**Keywords:** acidosis lactic, cannabis (marijuana), multiorgan toxicity, recreational cannabis, vomiting

## Abstract

The use of marijuana and cannabinoids is widespread throughout the United States, and these drugs can cause both acute and chronic medical disorders. The ingestion of edible products with uncertain content can cause accidental toxicity. The patient described in this case report ingested a large number of edible cannabinoid gummies. He developed a sequence of toxic states that included lethargy, agitation, vomiting, tachycardia, hypertension, acute respiratory failure, and elevated lactic acid levels. These clinical syndromes resolved over the next 24 hours with supportive intensive care unit management. The medical literature indicates that cannabis toxicity can cause acute lactic acidosis in some patients. This possibility needs to be considered when patients present to the emergency room with hypertension, tachycardia, and lactic acidosis

## Introduction

The use of marijuana and its chemical constituents continues to increase in the United States. The estimated cannabidiol (CBD) sales in Texas in 2023, for example, were approximately 8 billion dollars [1]. These products can be smoked, vaped, and ingested. Some individuals develop acute intoxication with psychosis, severe vomiting, tachycardia, or hypertension, or a combination of these presentations. In addition, a few cases have had significant lactic acidosis [2]. The patient described in this case report experienced a sequence of acute toxic disorders and lactic acidosis.

## Case presentation

A 29-year-old man in good health (BMI-30 kg/m2) with no chronic medical disorders presented with an unintentional overdose of CBD gummies. Emergency medical services were called when he reportedly vomited on himself and became unresponsive. Upon their arrival, he was very lethargic but then became combative en route and was given 400 mg of IM ketamine. He continued to be combative in the emergency department and started to have projectile vomiting, which led to intubation for airway protection. He was also tachycardic (Table 1).

**Table 1:** Vital signs

Hospital day	Time	Heart rate Beats/minute	Blood pressure mmHg	Respiratory rate^*^ Breaths/minute
1	19:50	165	128/68	20
1	20:50	138	137/74	20
1	21:05	115	137/81	20
1	22:15	96	96/58	20
1	23:00	75	97/53	20
2	00:00	71	116/68	20
2	01:00	74	120/71	20
2	02:00	70	94/60	20
2	3:00	76	101/51	15

His highest blood pressure during the initial hours of admission was 153/78 mmHg. He was successfully extubated less than 24 hours later. His urine drug screen was positive for marijuana (delta-9-tetrahydrocannabinol (THC)). His urine drug screen was negative for amphetamines and cocaine. Alcohol, acetaminophen, and salicylate levels were negative. His highest lactic acid level was 4.1 mmol/L (Table 2).

**Table 2:** Laboratory test * values in parentheses are the normal range for the test in our hospital; **CK - 127 U/L; ***ND - not done HCO_3_: bicarbonate; BUN: blood urea nitrogen; PCO2: partial pressure of carbon dioxide; CK: ; Cr

Hospital day	Sodium Meq/L (136-145)^*^	Potassium Meq/L (3.5-5.1)	Chloride Meq/L (97-107)	HCO_3_ Meq/L (20-30)	Anion gap Meq/L (4-12)	BUN/Cr mg/dL/ mg/dL (6-20/0.7-1.3)	Lactic* acid mmol/L (0.5-2.2)	Glucose mg/dL (65-115)	pH/PCO_2_ (7.3-7.5/30-50)
1**	138	5.0	104	20	14	12/1.1	3.2	153	7.34/43.3
2	137	3.9	103	19	15	13/1.1	4.1	126	7.42/32.6
3	140	3.8	106	24	10	14/1.1	2.4	101	ND***

After extubation, he was questioned about his overdose. He reported taking CBD gummy candies that had a brand name label. We attempted to find the exact product online to validate the dose but were unsuccessful. Despite this, the patient was confident that he took a total dose of 1600 mg of CBD, but he did not know the exact composition of these gummies, including the content of THC. He denied using CBD or using other THC products on a regular basis.

## Discussion

This patient presented with lethargy and a reduced level of consciousness and then developed agitation requiring sedation. This presentation seems consistent with delirium; he also had vomiting, tachycardia, and hypertension. His initial laboratory revealed a mixed acid-base disorder with mild acidemia secondary to lactic acidosis but no compensatory respiratory response. A literature review identified several cases of acute cannabis toxicity complicated by lactic acidosis.

Vo et al. reported information from a mass casualty event in which 12 children and 9 adults inadvertently ingested products containing THC [2]. The children had abnormal visual symptoms, dizziness, lethargy, tachycardia (n=10, 83%, range 102-181 beats per minute), tachypnea (n=6, 50%, range 22-30 breaths per minute), and hypertension (n=8, 66%, range 127-157 mmHg systolic). Seven pediatric patients (58%) had elevated lactate levels (2.1-4.5 mmol/L), and five (42%) had leukocytosis. Only one adult (11%) had elevated lactate levels. Both adults and children recovered within 12 hours; some patients received intravenous fluids. The authors suggest that these laboratory results might have reflected the physiologic effects of cannabinoids with increased adrenergic, serotoninergic, and dopaminergic stimulation. Alternatively, they may have had increased anaerobic muscle metabolism from increased work of breathing or other muscular activity. These cases demonstrate that toxic effects can occur in both children and adults with accidental ingestion and cause adrenergic hyperactivity and lactic acidosis.

Gajjala et al. presented an abstract at a Society of Critical Care Medicine meeting in 2022 that reported the case of a 26-year-old man who ingested ice cream containing cannabis [3]. This patient developed altered mental status, was febrile (101.9°F), and had a heart rate of 156 beats per minute. Laboratory tests reveal an elevated lactate level at 4 mmol/L, and his urine drug screen was positive for THC. He recovered with supportive measures. Bass and Linz reported a 56-year-old man with no history of substance abuse or medical disorders [4]. He was found by coworkers with bizarre behavior, vomiting, and slurred speech. He had purchased CBD gummies for pain and anxiety relief. Three hours after presentation, his heart rate was 47 beats per minute, his respiratory rate was 8-12 breaths per minute, his blood pressure was 88/52 mmHg, and his O2 saturation was 78%. The patient eventually recovered with supportive care with intravenous fluids, oxygen, antiemetics, and continuous stimulation. His laboratory testing revealed an elevated lactate level at 2.4 mmol/L. Arterial blood gases included pH 7.30, partial pressure of carbon dioxide (PaCO2) 50.4 mmHg, and partial pressure of oxygen (PaO2) 83.3 mmHg. This case differs since the patient had bradycardia, bradypnea, and hypotension. Antill et al. reported a case of synthetic marijuana intoxication [5]. This patient was found unconscious at home with rapid, shallow breathing. His lactic acid level was 10.6 mmol/L. He had seizures after admission and required intubation and mechanical ventilation with a bicarbonate drip. These cases indicate that cannabis toxicity can cause both tachycardia and bradycardia in association with lactic acidosis.

Hopkins and Wilson reported a 69-year-old woman with a history of congestive heart failure who used marijuana daily [6]. She presented with shortness of breath, wheezing, vomiting, and abdominal pain and had tachycardia and tachypnea. Initial lab tests included a lactic acid level of 8.8 mmol/L with a repeat level of 13.6 mmol/L 6 hours after treatment. A workup for alternative explanations for lactic acidosis was negative. These authors concluded that she had persistent lactate lactic acidosis without shock, likely secondary to THC. This patient was older, had chronic heart disease, and developed lactic acidosis. The pathogenesis of lactic acidosis in this patient is likely more complicated than a simple ingestion with toxicity. Cummins and Croft reported a patient who had an alcohol use disorder and cirrhosis who developed significant shortness of breath while smoking marijuana more or less continuously on one day [7]. He had an elevated lactic acid level (25.6 mmol/L) and metabolic acidosis (bicarbonate (HCO3)- 5 mmol/L, anion gap-25 mmol/L) and required emergency dialysis with a good outcome. These two case reports demonstrate the variable clinical presentation associated with cannabis toxicity in patients with chronic medical disorders. Marijuana toxicity should be considered in patients who present with toxic syndromes and lactic acidosis, even if they have chronic medical disorders that potentially explain their presentation.

The cannabis plant has more than 125 phytocannabinoids [8]. The most studied compounds are delta-9-tetrahydrocannabinol (THC) and cannabidiol (CBD), and these chemicals can cause acute intoxication and can cause chronic use disorders. Tetrahydrocannabinol interacts with cannabinoid receptors types 1 and 2. Cannabinoid receptor 1 is found throughout the brain and in the myocardium, the vasculature endothelium, adipose tissue, liver, and reproductive organs. Cannabinoid receptor 2 is found on immune cells and endothelial cells. Cannabinoids also bind to other receptors, including transient receptor potential channels of type V1 and nuclear receptor peroxisomal proliferated-activated receptor gamma [9,10]. The exact effect(s) of activation of these receptors in patients presenting with toxic syndromes is uncertain. The pharmacology of cannabinoids depends on the route of administration; these drugs can cause several acute psychological and physiological effects. Psychological effects include euphoria, relaxation, and sedation, psychotic effects include hallucinations and delusions, and physical effects include impaired coordination, slurred speech, tachycardia, and orthostatic hypotension. Occasionally, these chemicals are associated with acute transient cardiac arrhythmias. Intoxication after inhalation occurs within minutes; intoxication after oral administration occurs within 30 minutes to 3 hours.

This patient in this report presented with a complex toxic syndrome that included sedation and then agitation. His initial vital signs included significant tachycardia and hypertension. He had elevated lactate levels that persisted for 3 days during his hospitalization. This likely represents type B lactic acidosis since he did not have hypoxemia or ischemia during his hospitalization [11,12]. This could be explained by aerobic glycolysis with increased production of lactate from pyruvate secondary to adrenergic hyperreactivity. However, he still had elevated levels on his third day of hospitalization when he was clinically stable. Alternatively, it could represent reduced oxidative phosphorylation with reduced metabolism of lactate to CO2 and H2O. He did not have any history of liver disease or renal disease, and this would not represent a generalized metabolic disorder associated with a chronic disease. Presumably, these elevated lactate levels reflect a direct inhibitory effect of CBD or other compounds in these gummies on oxidative phosphorylation. The patient had normal glucose levels during his hospitalization and did not have any defect in gluconeogenesis. Consequently, understanding the cause of elevated lactate levels in these patients would require a fairly sophisticated analysis of relevant metabolic pathways.

Reports from national poison control centers provide information about toxic effects associated with the use of marijuana and cannabinoids. Cao et al. analyzed 430 calls reported between January 2013 and December 2015 [13]. The most common age groups involved in these calls were children less than five years old and adolescents 13 to 19 years old. The most frequent symptoms included drowsiness/lethargy, tachycardia, agitation, and confusion. No deaths occurred. Eighty-six patients (20%) required intravenous fluids, 47 (11%) received benzodiazepines, and three patients required intubation. Miller et al. analyzed commercial CBD products, including oils, aqueous products, and various other products for CBD content [14]. Many samples were mislabeled and had either more CBD than advertised or less CBD than advertised. THC was detected in some of these products. Gidal et al. also analyzed CBD products and determined that the majority were mislabeled and that heavy metals, residual solvents, and pesticides were detected in many products [15]. Consequently, users cannot be certain of the products purchased at retail establishments. These may be mislabeled, may have THC, and may have other unexpected contaminants. This can cause significant difficulty in understanding the toxicity associated with CBD.

## Conclusions

Clinicians should consider the possibility of toxicity related to cannabinoid product ingestion when patients present to the emergency department with agitation, tachycardia, and anion gap metabolic acidosis associated with lactic acid levels. These patients usually recover with minimal medical intervention but need observation and often need intravenous fluids. The patient described in this case report ingested a large number of edible CBD products. He experienced a sequence of toxic states and required mechanical ventilation.
